# Pd-Based Bimetallic Electrocatalysts for Hydrogen Oxidation Reaction in 0.1 M KOH Solution

**DOI:** 10.3390/nano14060500

**Published:** 2024-03-11

**Authors:** Georgios Bampos, Symeon Bebelis

**Affiliations:** Department of Chemical Engineering, University of Patras, GR-26504 Patras, Greece; simeon@chemeng.upatras.gr

**Keywords:** hydrogen oxidation reaction, Pd-based electrocatalysts, alkaline medium, hydrogen binding energy, hydroxyl species binding energy, strain effect, oxophilic effect, rotating-disk electrode

## Abstract

A series of carbon black-supported 7.5 wt.% Pd-2.5 wt.% M/C (M: Ag, Ca, Co, Cu, Fe, Ni, Ru, Sn, Zn) electrocatalysts, synthesized via the wet impregnation method, and reduced at 300 °C, were compared in terms of their hydrogen oxidation reaction (HOR) activity in a 0.1 M KOH solution using the thin-film rotating-disk electrode technique. Moreover, 10 wt.% Pd/C and 10 wt.% Pt/C electrocatalysts were prepared in the same manner and used as references. The 7.5 wt.% Pd-2.5 wt.% Ni/C electrocatalyst exhibited the highest HOR activity among the Pd-based electrocatalysts, although it was lower than that of the 10 wt.% Pt/C. Its activity was also found to be higher than that of Pd-Ni electrocatalysts of the same total metal loading (10 wt.%) and reduction temperature (300 °C) but of different Pd to Ni atomic ratio. It was also higher than that of 7.5 wt.% Pd-2.5 wt.% Ni/C electrocatalysts that were reduced at temperatures other than 300 °C. The superior activity of this electrocatalyst was attributed to an optimum value of the hydrogen binding energy of Pd, which was induced by the presence of Ni (electronic effect), as well as to the oxophilic character of Ni, which favors adsorption on the Ni surface of hydroxyl species that readily react with adsorbed hydrogen atoms on neighboring Pd sites in the rate-determining step.

## 1. Introduction

In H_2_-fueled proton-exchange membrane fuel cells (PEMFCs), relatively low Pt loadings (≤0.05 mg_Pt_ cm^−2^) can result in high performance as it concerns the anodic H_2_ oxidation reaction (HOR) [1,2,3]. However, high Pt loadings (0.2–0.4 mg_Pt_ cm^−2^) are required for the electrocatalysts that are used for the cathodic O_2_ reduction reaction (ORR) due to the slow reaction kinetics of the latter [1,4,5]. The main obstacle to the application and mass production of PEMFCs is the high cost associated with the noble metal electrocatalysts used for achievement of high performance for the anodic and the cathodic reaction [4,5,6]. Anion alkaline exchange membrane fuel cells (AAEMFCs) can operate with electrocatalysts for the cathodic ORR having a lower Pt loading, due to the faster reaction kinetics of the ORR in an alkaline environment [4,7,8]. Non-noble metal (platinum group metal-free) electrocatalysts exhibit a similar or better performance for the ORR in alkaline media compared with Pt-based electrocatalytic systems [9,10,11,12,13]. However, the kinetics of the anodic HOR in alkaline media is ca. two orders of magnitude slower than in acidic media, thus requiring high Pt loadings in order to be efficiently catalyzed [3,14].

The design and synthesis of electrocatalytic systems that are active for the HOR in alkaline environment require the study of the HOR mechanism. The $H_{2}$ oxidation reaction in alkaline medium is described by the following equation [14]:

(1) $$H_{2}+2OH^{-}\to 2H_{2}O+2e^{-}$$

The HOR in alkaline medium may proceed via the Tafel–Volmer or Heyrovsky–Volmer mechanisms, which correspond to combinations of the following steps:

(2) $$\mathrm{Tafel}\;\mathrm{step}:\;H_{2}\to 2H_{ad}$$

(3) $$\mathrm{Heyrovsky}\;\mathrm{step}:\;H_{2}+OH^{-}\to H_{ad}+H_{2}O+e^{-}$$

(4) $$\mathrm{Volmer}\;\mathrm{step}:\;H_{ad}+OH^{-}\to H_{2}O+e^{-}$$

where $H_{ad}$ denotes adsorbed hydrogen atoms. Concerning the hydroxyl species participating in the HOR, they may be $OH^{-}$ species that are present in the electrolytic solution [3,15,16,17,18], as above, or adsorbed hydroxyls $OH_{ad}$, formed as follows [19,20,21]:

(5) $${OH}^{-}\to OH_{ad}+e^{-}$$

The simplest HOR kinetic model assumes that there is one rate-determining step (RDS) [15,22,23,24,25]. The Tafel slope has been used to identify the RDS of the HOR, with Tafel slope values of approximately 30 mV dec^−1^ and 120 mV dec^−1^ indicating hydrogen adsorption (Tafel step) and charge transfer (Heyrovsky or Volmer steps) as the RDS, respectively [3,15,18,26]. 

Concerning HOR activity in alkaline media, two activity descriptors have been proposed [14,16,19,27,28,29,30]. The first is the hydrogen binding energy (HBE) on the electrocatalyst [16,27], and the second is the oxophilicity of the electrocatalyst, as it affects the adsorption of hydroxyl species on the metal surface, in reference to the HOR mechanismthat involves $OH_{ad}$ (bifunctional role of the anode) [19,31]. Both HBE and metal oxophilicity may affect HOR in alkaline media, depending on the particular electrocatalyst and pH [16,19,27,32]. Achieving a balance between the adsorption of the hydrogen and hydroxyl species on the electrocatalytic surface can lead to an increase in HOR activity [20].

The above-mentioned different considerations regarding the HOR activity descriptors in alkaline environment increase the difficulty of the effective design of electrocatalytically active materials. Many research efforts have focused on the synthesis of noble metal-based electrocatalytic systems, studying the effect of the support [20,33,34,35,36,37], particle size [1,3,38,39,40,41] and structural characteristics [17,21,27,32,42,43,44,45,46,47,48,49], as well as on the development of noble metal-free electrocatalysts [29,50,51], which, however, exhibit lower activity than the noble metal-based electrocatalysts [50]. 

Towards the development of active electrocatalysts for HORs in alkaline environments with relatively reduced cost, a series of carbon black-supported Pd-M (M: Ag, Ca, Co, Cu, Fe, Ni, Ru, Sn, Zn) bimetallic electrocatalysts, with a 10 wt.% total metal loading and a Pd:M mass ratio equal to 3:1, were synthesized using the wet impregnation method, and their HOR activity in 0.1 M KOH solution was compared, using the thin-film rotating-disk electrode (RDE) technique. Most of the aforementioned Pd-based electrocatalytic systems have been tested by our research group for their HOR activity in an acidic medium [52], as well as their ORR activity in acidic [53] and alkaline media [54]. The most active electrocatalytic system was found to be Pd-Ni, for which the effect of the reduction temperature and Pd:Ni atomic ratio on HOR activity was also investigated. To the best of our knowledge, the present work is the first systematic study of HOR activity in 0.1 M KOH of Pd:M bimetallic electrocatalysts, accompanied by optimization of the most active bimetallic system (Pd:Ni). 

## 2. Materials and Methods

### 2.1. Synthesis of the Catalytic Powders

The carbon black-supported Pd-M (M: Ag, Ca, Co, Cu, Fe, Ni, Ru, Sn, Zn) catalytic powders were synthesized by employing the wet impregnation (w.i.) method, as described in detail elsewhere [53]. Briefly, appropriate amounts of metal precursors were diluted under continuous flow in triple-distilled water at room temperature, and then an appropriate amount of carbon black support (Vulcan XC72R, Cabot, Billerica, MA, USA) was added. The mixture was heated to 70 °C and was maintained at this temperature until complete evaporation of H_2_O. The resulting slurry was dried overnight at 110 °C, and the obtained powder was reduced. The metal precursors used for the w.i. synthesis were PdCl_2_, AgNO_3_, Ca(NO_3_)_2_·4H_2_O, CoCl_2_·6H_2_O, Cu(NO_3_)_2_·3H_2_O, FeCl_2_·6H_2_O, Ni(NO_3_)_2_·6H_2_O, SnCl_2_, Zn(NO_3_)_2_·6H_2_O, (NH_3_)_4_Pt(OH)_2_ and Ru(NO)(NO_3_)_3._ All were purchased by Alfa Aesar (Ward Hill, MA, USA), except for Ru(NO)(NO_3_)_3_, which was purchased by SERVA (Heidelberg, Germany). Details concerning their characteristics can be found elsewhere [53].

The synthesized 7.5 wt.% Pd-2.5 wt.% M/C catalytic samples that were initially screened for their HOR activity in 0.1 M KOH were reduced at 300 °C under H_2_ flow for 2 h. For the most active Pd-Ni bimetallic system, which turned out to be 7.5 wt.% Pd-2.5 wt.% Ni/C, catalytic powders were reduced at four different temperatures, namely 200, 300, 450, and 600 °C, and the corresponding electrocatalysts were compared for their HOR activity. In addition, the effect on HOR activity of the Pd:Ni ratio was studied for a series of bimetallic Pd-Ni catalytic samples of 10 wt.% total metal loading, synthesized in the same manner and reduced at 300 °C.

### 2.2. Physicochemical Characterization of the Catalytic Powders

The specific surface area (SSA) of the tested catalytic powders was measured via N_2_ physisorption at liquid nitrogen temperature (−196 °C) on a Micromeritics (Norcross, GA, USA) Gemini III 2375 analyzer, employing the BET method. The X-ray diffraction (XRD) technique was used to characterize the crystallographic phases present in the synthesized catalytic powders and determine their structural characteristics. The XRD measurements were carried out on a Philips (Malvern Panalytical, Malvern, UK) PW 1830/40 X-ray diffractometer, and the identification of the phases was performed using the JCPDS data files. Details on the aforementioned instrumentation and characterization procedure can be found elsewhere [52,53,54,55]. 

### 2.3. Working Electrode Preparation and Electrochemical Characterization

The determination of the electrochemically active surface area (ECSA), as well as the assessment of the HOR activity in 0.1 M KOH electrolyte solution, was performed in a three-electrode system at room temperature (ca. 20 °C) [54]. Details concerning the experimental apparatus and instrumentation can be found in previous works of our group [52,53,54,55]. Each tested electrocatalyst was deposited in the form of a thin film on a rotating glassy carbon electrode (geometric surface area of 0.196 cm^2^) by pipetting a suspension of the electrocatalyst in a mixture of triple-distilled water and 2-propanol, whereas a mixture of 5 wt.% Nafion solution, 2-propanol, and triple-distilled water was used as binding agent [52,53,54,56].

The loading of the electrocatalyst was adjusted at 15 μg_met_ cm^−2^ after previous relevant calibration [54,56]. The potential of the reference electrode, which was a Ag/AgCl (3 M KCl) electrode, was equal to 0.960 V vs. RHE as measured in $H_{2}$-saturated 0.1 M KOH and ca. 20 °C [54]. Prior to each measurement, the deposited electrocatalytic film (working electrode) was activated by cycling its potential from 0.8 V to −1 V vs. Ag/AgCl (1.76 V to −0.04 V vs. RHE) in He-saturated electrolytic solution. The ECSA was determined using the CO stripping method, as detailed elsewhere [54]. 

The HOR measurements were carried out in $H_{2}$-saturated 0.1 M KOH solution, scanning the potential of the electrocatalysts from −1 V to −0.2 V vs. Ag/AgCl (−0.04 V to 0.76 V vs. RHE) at a scan rate equal to 5 mV s^−1^ and using a rotation rate of 3000 rpm. The electrolyte resistance that was used to calculate the ohmic drop-free applied potential, equal to ca. 41 Ω, was determined by means of electrochemical impedance spectroscopy (frequency range: 100 kHz–10 mHz, stimulus amplitude: 10 mV) [54]. 

It should be noted that the experiments were performed in 0.1 M KOH aqueous solution mainly to facilitate comparison with the results of previous studies. However, differences in HOR activity have been reported for 0.1 M aqueous solutions of different alkali hydroxides (LiOH, NaOH, KOH, and CsOH), largely associated with non-covalent interactions between the hydrated cations and the adsorbed reacting species [57,58]. It is also worth noting that the solubility and diffusion coefficient of hydrogen in alkaline electrolytes increase with decreasing molar concentration [59]. 

## 3. Results and Discussion

### 3.1. Effect of Transition Metal M on the HOR Activity of Pd-M Electrocatalysts

The effect of metal M in Pd-M electrocatalysts on their HOR activity in 0.1 M KOH was investigated using a series of 7.5 wt.% Pd-2.5 wt.% M/C (M: Ag, Ca, Co, Cu, Fe, Ni, Ru, Sn Zn) catalysts that were synthesized via the w.i. method in the form of powders and reduced at 300 °C under $H_{2}$ flow. Monometallic 10 wt.% Pd/C and 10 wt.% Pt/C catalysts synthesized using the same method were employed as reference materials. The notation, the chemical composition, the SSA, and the average metal particle size of the above materials are shown in Table 1. The SSA and the average metal particle size for these samples, as determined via the BET method and transmission electron microscopy (TEM), respectively, have also been presented in earlier works of our group [52,53,54]. The deposition of the metal phase on carbon black yielded a significant reduction of the SSA values (Table 1), which was attributed to pore blockage of the carbon black support [53]. The highest SSA among the Pd-based materials was exhibited by 7.5 Pd-2.5 Ag (171 m^2^ g^−1^), followed by 10 Pd (143 m^2^ g^−1^) and 7.5 Pd-2.5 Sn (141 m^2^ g^−1^). The lowest SSA was measured for the 7.5 Pd-2.5 Ca (62 m^2^ g^−1^) catalytic powder. Regarding the average metal particle size, the smallest one was observed for the 10 Pt catalyst (2.1 nm). Among the Pd-based samples, the smallest average particle size was observed for 7.5 Pd-2.5 Ni (3 nm) and the largest for 7.5 Pd-2.5 Ru (13 nm), followed in descending order by 7.5 Pd-2.5 Sn (8.5 nm). The XRD spectra of the catalytic powders have been presented in previous works of our group [52,53,54]. As it concerns the Pd-based samples, characteristic peaks attributed to Pd were observed in all XRD spectra, whereas in the case of 7.5 Pd-2.5 Zn, two additional peaks attributed to PdZn alloy were also detected. For the 7.5 Pd-2.5 Ag powder, peaks corresponding to Ag were also present in the XRD spectrum. 

Table 1 also shows the ECSA_CO_ values of the corresponding electrocatalysts, as determined by the CO stripping method. The CO stripping curves used for the ECSA determination have been presented in previous work [54]. The highest ECSA_CO_ was exhibited by the 10 Pd electrocatalyst (20.4 m^2^ g^−1^), followed by 10 Pt (15 m^2^ g^−1^) and 7.5 Pd-2.5 Ag (14 m^2^ g^−1^), whereas the lowest ECSA_CO_ was exhibited by the 7.5 Pd-2.5 Co electrocatalyst (3.9 m^2^ g^−1^).

**Figure 1:**
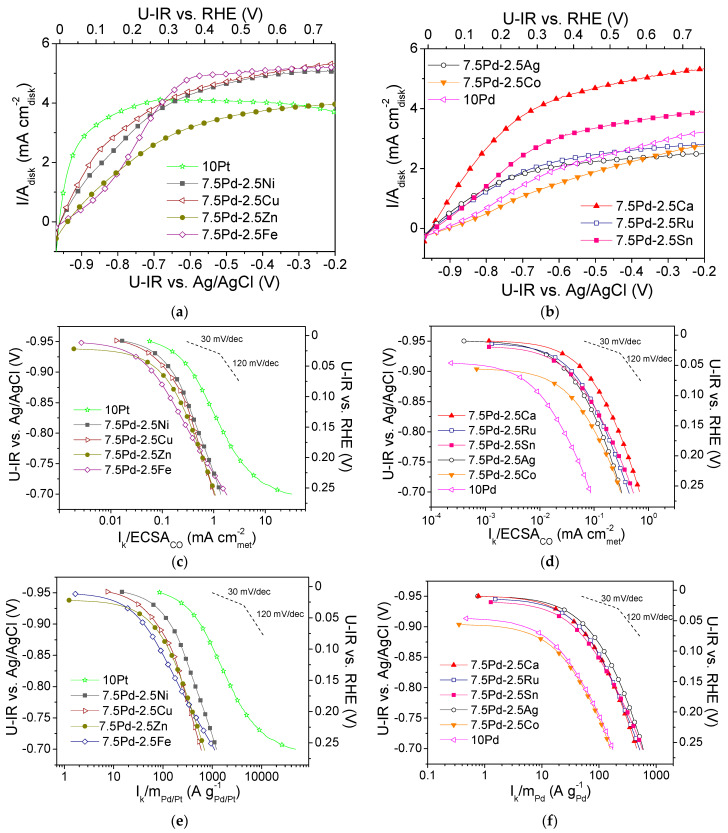
Polarization curves for the (**a**) 10 Pt, 7.5 Pd-2.5 Cu, 7.5 Pd-2.5 Fe, 7.5 Pd-2.5 Ni, 7.5 Pd-2.5 Zn and (**b**) 10 Pd, 7.5 Pd-2.5 Ag, 7.5 Pd-2.5 Ca, 7.5 Pd-2.5 Co, 7.5 Pd-2.5 Ru, 7.5 Pd-2.5 Sn electrocatalysts, obtained in 0.1 M KOH solution saturated with $H_{2}$, at room temperature and 3000 rpm, by scanning the potential of the electrocatalyst at a rate of 5 mV s^−1^. Mass-transfer corrected Tafel plots for the HOR with the kinetic current *I_k_* normalized with respect to ECSA_CO_ (**c**,**d**) and the Pt or Pd mass of the electrocatalyst (**e**,**f**). Potential values (U-IR) are ohmic drop corrected.

Figure 1a,b show polarization curves of the tested electrocatalysts that were obtained in $H_{2}$-saturated 0.1 M KOH solution at 3000 rpm. The measured current *I* is normalized with respect to the geometric surface area of the electrocatalyst, *A_disk_*. Mass-transfer limiting current (*I_lim_*) was observed, with the limiting current density (*i_lim_* = *I_lim_*/*A_disk_*) ranging from ca. 2 to 5 mA ${\mathrm{cm}}_{\mathrm{disk}}^{-2}$. The kinetic current *I_k_* (i.e., the intrinsic HOR rate) was calculated using the polarization curves via the equation [60]:

(6) $$\frac{1}{I}=\frac{1}{I_{k}}+\frac{1}{I_{lim}}$$

The normalization of the calculated kinetic current to the *ECSA_CO_* and the mass of the noble metal (Pd or Pt) mass resulted in the specific activity (S.A.) and mass activity (M.A.) for HOR, respectively. In Figure 1c–f, the S.A. and the M.A. of the tested electrocatalysts versus the ohmic drop-free applied potential, *U-IR*, are presented in the form of semilogarithmic plots (Tafel plots). The S.A. and M.A. of the most active electrocatalysts are shown in Figure 1c and Figure 1e, respectively. The most active electrocatalyst in terms of both S.A. and M.A. was 10 Pt (Figure 1c,e). Among the Pd-based electrocatalysts, the highest activity was exhibited by 7.5 Pd-2.5 Ni (Figure 1c,e), followed by the 7.5 Pd-2.5 Cu electrocatalyst, which exhibited similar S.A. (Figure 1c) but lower M.A. (Figure 1e). The 7.5 Pd-2.5 Zn and 7.5 Pd-2.5 Fe electrocatalysts exhibited similar specific activities with the 7.5 Pd-2.5 Cu electrocatalyst (Figure 1c), followed in descending order by 7.5 Pd-2.5 Ca and by the 7.5 Pd-2.5 Sn, 7.5 Pd-2.5 Ru and 7.5 Pd-2.5 Ag electrocatalysts, which had similar specific activities (Figure 1d). The lowest S.A. was exhibited by the 7.5 Pd-2.5 Co and 10 Pd electrocatalysts (Figure 1d). In terms of M.A., the Pd-based electrocatalysts followed the descending order (Figure 1e,f): 7.5 Pd-2.5 Ni > 7.5 Pd-2.5 Cu and 7.5 Pd-2.5 Zn > 7.5 Pd-2.5 Fe > 7.5 Pd-2.5 Ag > 7.5 Pd-2.5 Ru, 7.5 Pd-2.5 Ca and 7.5 Pd-2.5 Sn > 7.5 Pd-2.5 Co > 10 Pd. 

**Figure 2:**
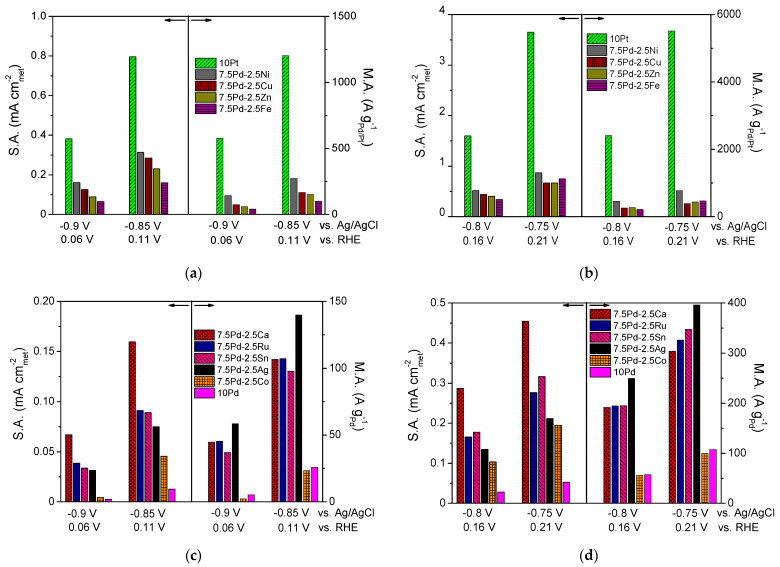
Comparison of the specific activity (S.A.) and mass activity (M.A.) of the (**a**,**b**) 10 Pt, 7.5 Pd-2.5 Cu, 7.5 Pd-2.5 Fe, 7.5 Pd-2.5 Ni, 7.5 Pd-2.5 Zn and (**c**,**d**) 10 Pd, 7.5 Pd-2.5 Ag, 7.5 Pd-2.5 Ca, 7.5 Pd-2.5 Co, 7.5 Pd-2.5 Ru, 7.5 Pd-2.5 Sn electrocatalysts at selected potentials (ohmic drop-free). Conditions: 0.1 M KOH solution saturated with $H_{2}$ and room temperature.

From the above observations, it can be concluded that the addition of a second metal M in the active metal phase, accompanied by a corresponding decrease of the Pd content, resulted in an increase in the HOR activity of the Pd-based electrocatalytic systems in 0.1 M KOH. Over almost the entire examined potential range, the 10 Pd electrocatalyst exhibited the lowest HOR activity. The presence of Ni in the metal phase seems to lead to a more pronounced increase of the HOR electrocatalytic activity compared to that for the other transition metals combined with Pd. However, the HOR activity of the tested 7.5 wt.% Pd-2.5 wt.% M/C electrocatalysts, although higher than that of 10 wt.% Pd/C, was lower than the activity of 10 wt.% Pt/C. To facilitate the comparison of the intrinsic HOR activity of the tested electrocatalysts, bar graphs depicting the S.A. and M.A. at selected ohmic drop-free applied potentials are presented in Figure 2. As shown in the figure, the 10 Pt and 7.5 Pd-2.5 Ni electrocatalysts exhibited the highest S.A. and M.A. over the entire examined potential range (Figure 2a,b). The 7.5 Pd-2.5 Cu electrocatalyst was the second most active one among the Pd-based electrocatalysts for applied potentials equal to −0.9 V and −0.85 V vs. Ag/AgCl (Figure 2a), corresponding to the low overpotential region. The above activity order was also observed for −0.8 V vs. Ag/AgCl (Figure 2b). Further increasing of the applied potential to −0.75 V vs. Ag/AgCl resulted in changes in both the S.A. and M.A. order, with the activity of 7.5 Pd-2.5 Fe and 7.5 Pd-2.5 Zn exceeding that of 7.5 Pd-2.5 Cu (Figure 2b). The S.A. and M.A. for HOR of the less active electrocatalysts at four selected applied potentials are shown in Figure 2c,d. As shown in the figures, the highest S.A. among the less active electrocatalysts was exhibited by 7.5 Pd-2.5 Ca, followed by 7.5 Pd-2.5 Ru and 7.5 Pd-2.5 Sn in S.A. descending order in the low overpotential region (Figure 2c), while at higher overpotentials this S.A. order was reversed (Figure 2d). The 7.5 Pd-2.5 Ag electrocatalyst had a lower S.A. than the aforementioned electrocatalysts; however, its M.A. exceeded that of the Pd-Ca, Pd-Ru and Pd-Sn electrocatalysts. The lowest HOR activity among all tested electrocatalysts was exhibited by 10 Pd and 7.5 Pd-2.5 Co (Figure 2). 

**Figure 3:**
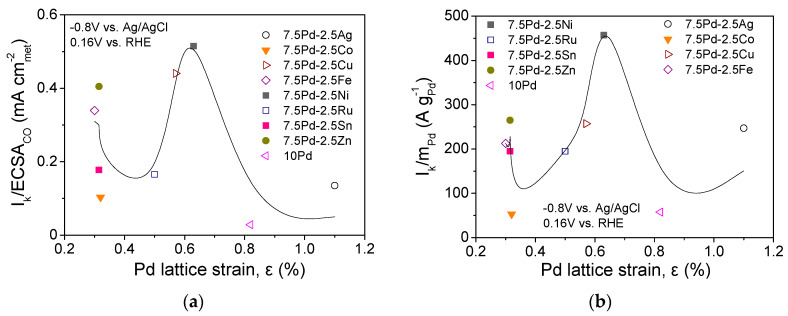
Correlation between the Pd lattice strain and (**a**) HOR specific activity and (**b**) HOR mass activity of the tested Pd-based electrocatalysts (Table 1) in 0.1 M KOH solution saturated with $H_{2}$ and room temperature.

The lattice strain of the metal electrocatalysts may affect the bonding of the adsorbed species participating in HOR in alkaline medium [49,61,62], similar to ORR [28,54,63,64]. Figure 3 shows the correlation between the HOR specific (Figure 3a) and mass activity (Figure 3b) at −0.8 V vs. Ag/AgCl (0.16 V vs. RHE) and the Pd lattice strain, *ε*, as calculated using the peak at 2*θ* = 68°, which corresponds to the (220) plane of the Pd fcc structure, via the following equation [54,65]:

(7) $$\varepsilon \left(\%\right)=\frac{\beta_{hkl}}{4\;tan\theta }100$$

where $\beta_{hkl}$ denotes the full width at half maximum of the considered XRD peak (in radians) [54,65]. As shown in the figure, both the S.A. and the M.A. exhibit a maximum for a Pd lattice strain close to 0.65%, which corresponds to the most active 7.5 Pd-2.5 Ni and 7.5 Pd-2.5 Cu electrocatalysts. This implies that the superior activity of 7.5 Pd-2.5 Ni may be partly explained by an optimum Pd lattice strain associated with an optimum adsorption strength of the adsorbates that are involved in the HOR mechanism [49,61,62]. A similar volcano-plot correlation between electrocatalytic activity and Pd lattice strain has been observed by our group for ORR in 0.1 M KOH over the same series of electrocatalysts (excluding 7.5 Pd-2.5 Ru) [54]. Interestingly, the optimum value of Pd lattice strain for ORR in 0.1 M KOH was close to 0.6%, corresponding to 7.5 Pd-2.5 Cu [54], which was the second most active Pd-based electrocatalyst for HOR in the present work. 

Hydrogen adsorption strength on Pd and Pt surfaces is relatively strong [49,61,62,66]. This can be explained on the basis of the d-band model [67,68], taking into account that since their d-band is more than half-filled [67], the center of the d-band is close to the Fermi level, so most of the anti-bonding states of adsorbed hydrogen are expected to be above the Fermi level and, being unoccupied, will not weaken the chemisorptive bond [68]. A common strategy to weaken the HBE on Pt or Pd is combining them with another transition metal [29,49,61,62,66]. The observed superior HOR activity in 0.1 M KOH of Pt-Ru/C over Pt/C nanoparticles has been mainly attributed to the observed contraction of the Pt lattice due to incorporation of Ru atoms and the resulting decrease of the Pt-Pt distance which affects the electronic properties of the bimetallic electrocatalyst (lattice strain effect) causing a downshift of the Pt d-band center and a concomitant weakening of adsorbed hydrogen binding energy on Pt in the rate-determining step [61,62]. This beneficial effect of alloying Pt with Ru on HOR activity has been reported to be optimized by tuning the near-surface composition of the Pt-Ru alloy for the same bulk composition via controlled thermal treatment [61] or by controlling the alloying degree via changing the synthesis temperature [62]. Scofield et al. [49] have reported that the HOR activity of PtM alloy nanowires (M: Fe, Co, Ru, Cu, Au) in 0.1 M KOH, based on exchange current density, followed the same trend as the theoretically calculated corresponding HBE values, with the exception of PtCu, for which however the reduced HOR activity compared to Pt was explained by formation of copper oxide during testing at high potentials and resulting blocking of Pt active sites. On the basis of structural, XPS, and electrochemical characterization, they attributed the observed higher HOR activity of the PtCo, PtFe, and Pt-Ru nanowires over the Pt nanowires to an electronic effect in which both a lattice strain effect, as described above, and a ligand effect (change in the overlap of the d-bands of both Pt and the second metal, resulting from electron density transfer) synergistically contribute to decrease HBE [49]. Wang et al. [27] have also attributed the enhanced HOR activity of Pt-Ru/C over Pt/C in 0.1 M KOH to an electronic effect causing a decrease of the HBE, reporting the effect of different oxophilicity of Pt and Ru as insignificant on the basis of CO stripping experiments. Similar conclusions were drawn by Lu and Zhuang [42] for the enhanced HOR activity in 0.1 M of surface-controlled PtNi/C over Pt/C nanoparticles. They attributed this mainly to a reduction of HBE induced by alloying, as reflected in the appearance of H_upd_ peaks at lower potential for PtNi/C compared to Pt/C, due to lowering of the Pt d-band center (geometric and electronic effect) [69,70,71], considering the role of OH adsorption much less significant and proposing an HOR mechanism not involving $OH_{ad}$. Alia et al. [21,32] investigated the HOR activity in an alkaline medium of Pt-Cu and Pd-Cu bimetallic systems. They found that Pt- and Pd-coated Cu nanowires exhibited significantly higher HOR activity than carbon-supported Pt. This was mainly attributed to the compressive strain effect on Pt or Pd caused by the presence of Cu, resulting in a downshift of the Pt or Pd d-band center relative to the Fermi level and concomitant decrease in HBE, but also to enhancement of the hydroxyl adsorption due to the oxophilic properties of Cu. 

Although HBE has been widely recognized as a key descriptor of HOR activity in alkaline media [29,30,49,62,66], the adsorbed hydroxyl species binding energy (OHBE), which is affected by the oxophilicity of the electrocatalytically active metal phase, has also been reported to play an important role in determining HOR activity (bifunctional mechanism) [19,29,30,72,73,74,75]. Based on this, enhancement of HOR activity in alkaline media is expected by combining Pt or Pd with oxophilic metals (such as Ni, Co, Ru, etc.), which exhibit higher OHBE and, thus, enhance the adsorption of hydroxyls [72,73,74]. Li et al. [75] verified the bifunctional HOR mechanism on Pt-Ru/C in 0.1 M KOH by combining electrochemical and in situ spectroscopic (XANES) data, which provided evidence for dynamic co-existence of $OH_{ad}$ and $H_{ad}$ on Ru in the HOR region and confirmed the promoting effect of $OH_{ad}$ on Ru in facilitating the oxidation of $H_{ad}$ on adjacent Pt sites in the RDS. Wang et al. [74] used in situ electrochemical surface-enhanced Raman spectroscopy (SERS) to confirm the presence of $OH_{ad}$ on the surface of PtNi alloy shell-Au core nanoparticles during HOR in 0.1 M KOH, which, according to DFT calculations, are adsorbed on Ni-Ni bridge sites and react readily with adsorbed atomic hydrogen atoms on neighboring Pt sites in the RDS, with concomitant enhancement of the HOR activity. Alesker et al. [20], in their study of HOR in an alkaline medium over a Pd-Ni electrocatalyst consisting of Pd nano-islands supported on Ni nanoparticles, attributed the observed superior HOR activity of Pd-Ni compared to monometallic Pd to the enhanced adsorption of hydroxyl species onto the oxophilic Ni which can react at a high rate with adsorbed hydrogen atoms on Pd neighboring sites (or even more distant Pd sites, due to fast surface diffusion of $H_{ad}$ on Pd islands), in contrast to HOR on bare Pd where hydroxyl species should be supplied directly from the electrolyte. As they found no evidence of alloying between the two metals, they considered that in the studied system, Ni is not expected to have a significant electronic effect on HBE on Pd, irrespective of the fact that the surface site composition and energetics may be different from those in monometallic Pd and/or Ni electrocatalysts [20]. Similar conclusions concerning the promoting effect of the oxophilic character of Ni for HOR in alkaline medium have been drawn by Bakos et al. [44] who studied HOR in 0.1 M KOH on Pd nanoscale-coated Ni electrodes and reported an increase in HOR activity with increasing Pd coverage up to an optimum value 17%, at which the diffusion limit for HOR is reached. Considering the synthesis method (spontaneous electrolytic deposition of Pd) and the atomic force microscope characterization, they concluded that the surface of the Pd-Ni electrocatalysts most likely consisted of Pd islands on Ni with limited metal interdiffusion, which makes any electronic effects less possible and highlights the dominant promoting role of the oxophilicity of Ni on HOR in this system by enabling the presence of $OH_{ad}$ in the vicinity of $H_{ad}$ on Pd sites [44]. 

The metal particle size has also been reported to affect HOR activity [1,29,38,40,41,76,77]. Zheng et al. [1] have found an increase in HOR activity in both 0.1 M HClO_4_ and 0.1 M KOH of carbon-supported Pd nanoparticles with increasing particle size from 3 to 19 nm, beyond which (up to 42 nm) no further increase in activity (in terms of exchange current density normalized to ECSA) was observed. This enhancement of HOR activity was attributed to an increase in the percentage of the Pd sites of smaller HBE, as revealed by cyclic voltammetry, caused by a redistribution of surface facets in combination with the possible structure sensitivity of HOR [1,29]. On the contrary, Wang and Abruña [77] reported that a Rh/C electrocatalyst with an average particle size of ca. 2 nm was more active for HOR in 0.1 M KOH than another one with an average particle size of ca. 5 nm, prepared by the same impregnation method but a different precursor. A volcano-type dependence of HOR activity in 0.1 M NaOH (in terms of specific and mass exchange current density) on Ru particle size has been reported by Ohyama et al. [38] for carbon-supported Ru nanoparticles in the range of 2 to 7 nm. The observed activity maximum for 3 nm Ru nanoparticles, higher than that of a Pt/C (TKK) commercial electrocatalyst, was associated with the presence of an optimum fraction of coordinatively unsaturated long-bridged Ru atoms on the nanoparticle surface, which allows the adsorption of hydrogen in the presence of surface oxygenated species, the latter being favored by the oxophilicity of Ru [38]. Also, carbon-supported Ru nanoparticles of ca. 3 nm diameter showed better performance than Ru nanoparticles of ca. 11 nm in diameter as anode electrocatalysts in AAEMFC testing [41]. Zheng et al. [40] reported a decrease in the HOR activity in 0.1 M KOH of a series of Ir/C electrocatalysts with decreasing Ir particle size in the range of 2–7 nm when using the exchange current density normalized with respect to the total ECSA as an activity indicator. This decrease was accompanied by a decrease in the population of sites with smaller HBE, as determined by analysis of cyclic voltammograms in the H_upd_ desorption region. On the contrary, no change in particle size was observed when the exchange current density was normalized to the part of ECSA corresponding to the sites of the lowest HBE, which indicates that those sites are the most active for HOR [40]. 

Based on the above, the observed differences in the intrinsic HOR activity in 0.1 M KOH of the tested electrocatalysts can be mainly associated with electronic and oxophilic effects, although differences in particle size and surface morphology may also need to be considered. The superior HOR activity of the 7.5 Pd-2.5 Ni electrocatalyst among the tested Pd-based electrocatalysts (Figure 1 and Figure 2) could be partly attributed to an induced optimum Pd lattice contraction of ca. 0.65%, which results in the lowering of HBE on Pd at an optimum value favoring HOR on the basis of its mechanism, and partly to the higher oxophilicity of Ni compared to Pd [78], which favors the adsorption of hydroxyl species on Ni facilitating their reaction with $H_{ad}$ on neighboring Pd sites, thus increasing the HOR rate. The metal particle size of 7.5 Pd-2.5 Ni, which was the smallest among those measured for the Pd-based catalysts, may also be associated with a more favorable surface nanostructure for HOR, given the structure sensitivity of this reaction [1,38]. For 7.5 Pd-2.5 Cu, the second more active Pd-based electrocatalyst in terms of specific activity (Figure 1a), as well as mass activity (Figure 1c) at low overpotentials, the Pd lattice contraction is very close to that for 7.5 Pd-2.5 Ni (Figure 2), while Cu and Ni have the same oxophilicity (lower than that of Ca, Sn, Co, Ru and equal to that of Ag and Zn) [78], possibly corresponding to an optimized adsorption of hydrogen on Pd and hydroxyl species on Cu or Ni that favoring HOR. It should also be noted that for 7.5 Pd-2.5 Ni and 7.5 Pd-2.5 Cu the ratios of Ni to Pd atoms and Cu to Pd atoms on the catalyst surface were found by XPS to be almost three times and two times higher, respectively, than those calculated for the nominal composition [54], in contrast to the 7.5 Pd-2.5 Zn, 7.5 Pd-2.5 Sn and 7.5 Pd-2.5 Ag for which atomic ratio of the two metals on the surface was close to the nominal [52,54]. This enrichment of the catalyst surface with the oxophilic Ni and Cu may also promote HOR by favoring the interaction of these metals with Pd and the reaction between $OH_{ad}$ and $H_{ad}$ on neighboring sites. 

### 3.2. Effect of Reduction Temperature of 7.5 wt.% Pd-2.5 wt.% Ni/C Catalyst on the HOR Activity

The effect of reduction temperature on the HOR activity was studied for the 7.5 Pd-2.5 Ni electrocatalyst, which was the most active among the tested Pd-M electrocatalysts (Section 3.1). Different samples of this catalyst were reduced at four different temperatures, namely 200, 300, 450, and 600 °C under $H_{2}$ flow for 2 h, and were characterized as it concerns their physicochemical characteristics via ${Ν}_{2}$ physisorption (BET method) and XRD (Section 2.2). Their HOR electrocatalytic activity in 0.1 M KOH was assessed using the thin-film RDE technique (Section 2.3). 

Table 2 shows the notation, the reduction temperature, the SSA values, the Pd crystallite size, as calculated from XRD data, and the ECSA_CO_ values, as calculated using the CO stripping method [54]. The SSA values ranged from 98 m^2^ g^−1^ (7.5 Pd-2.5 Ni_200) to 134 m^2^ g^−1^ (7.5 Pd-2.5 Ni_600). Increasing the reduction temperature yielded an increase in the SSA. Specifically, the SSA increased from 98 m^2^ g^−1^ to 118 m^2^ g^−1^ by increasing the reduction temperature from 200 to 300 °C, while a further increase of the reduction temperature to 450 °C did not significantly affect the SSA (120 m^2^ g^−1^). By increasing the reduction temperature up to 600 °C, the SSA increased further to 134 m^2^ g^−1^.

**Figure 4:**
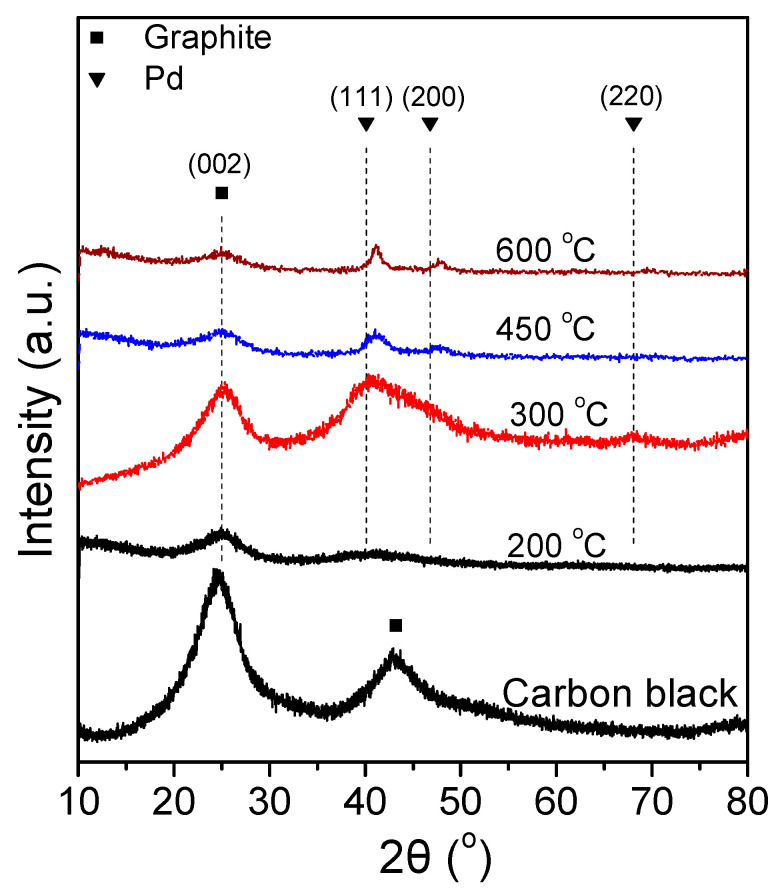
X-ray diffraction patterns of 7.5 Pd-2.5 Ni electrocatalyst powders reduced at 200, 300, 450, and 600 °C, as well as of the carbon black support (Vulcan XC72R).

Figure 4 shows the XRD spectra of the 7.5 wt.% Pd-2.5 wt.% Ni/C samples that were reduced at different temperatures. The XRD spectra of the 7.5 Pd-2.5 Ni_450 and 7.5 Pd-2.5 Ni_600 powders are characterized by diffraction peaks located at 2*θ* equal to 40.3°, 46.6°, and 68°, attributed to facets (111), (200) and (220), respectively, of the Pd fcc crystalline structure (JCPDS Card No. 46-1043). A small shift towards higher diffraction angles of the crystallographic peaks corresponding to Pd is an indication of alloy formation. However, to identify the type of a formed alloyed phase, such a shift should be accompanied by the appearance of crystallographic peaks corresponding to the alloyed compound. Therefore, even if alloy phases are formed in these samples, they are not present at a level detectable by the XRD method.

The average Pd crystallite size of the tested samples was calculated via the Scherrer equation [79] from the XRD peak located at 2*θ* = 40.3°. The largest average crystallite size was exhibited by 7.5 Pd-2.5 Ni_600, followed in descending order by the 7.5 Pd-2.5 Ni_450, 7.5 Pd-2.5 Ni_300, and 7.5 Pd-2.5 Ni_200 samples. Increasing the reduction temperature from 300 °C to 450 °C did not significantly affect the average Pd crystallite size. However, it was notably increased from 3.2 nm (7.5 Pd-2.5 Ni_450) to 7.9 nm (7.5 Pd-2.5 Ni_600) by further increase of the reduction temperature to 600 °C. Presumably, the increase in the reduction temperature results in sintering of the metal crystallites, thus increasing their size.

**Figure 5:**
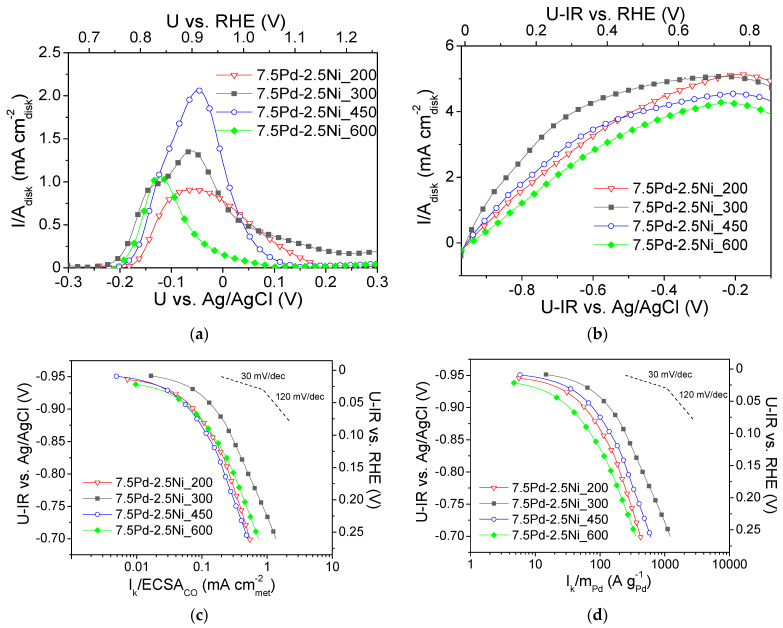
(**a**) CO stripping linear sweep voltammograms obtained in He-purged 0.1 M KOH solution at room temperature with a potential scan rate of 50 mV s^−1^ after previous adsorption of CO at −0.7 V vs. Ag/AgCl for 15 min; (**b**) Polarization curves obtained in 0.1 M KOH solution saturated with $H_{2}$, at room temperature and 3000 rpm, by scanning the potential of the electrocatalyst at a rate of 5 mV s^−1^; Mass-transfer corrected Tafel plots for the HOR in $H_{2}$ saturated 0.1 M KOH solution at room temperature with the kinetic current *I_k_* normalized with respect to ECSA_CO_ (**c**) and the Pd mass (**d**) for the 7.5 Pd-2.5 Ni electrocatalysts reduced at 200, 300, 450 and 600 °C (Table 2). U-IR denotes ohmic drop corrected potential.

Figure 5a shows CO stripping curves obtained in He-purged 0.1 M KOH electrolyte solution after previous saturation with CO [54] for the four 7.5 Pd-2.5 Ni samples, which were reduced at different temperatures (Table 2). As shown in Table 2, increasing the reduction temperature from 200 to 450 °C increased the ECSA_CO_ (determined via CO stripping, Figure 5a) from 5.8 m^2^ g^−1^ (7.5 Pd-2.5 Ni_200) to 9 m^2^ g^−1^ (7.5 Pd-2.5 Ni_450), while a further increase in the reduction temperature to 600 °C yielded a significant decrease of the ECSA_CO_ value to 3.6 m^2^ g^−1^ (7.5 Pd-2.5 Ni_600). This decrease could be associated with the observed increase in the average Pd crystallite size (Table 2), which is expected to result in a decrease of the Pd dispersion on the carbon black support and, in turn, a decrease in the electrochemically active area.

Figure 5b shows the polarization curves obtained in $H_{2}$ saturated 0.1 M KOH solution at 3000 rpm for the four tested 7.5 Pd-2.5 Ni electrocatalysts (Table 2). The values of the limiting current density ranged from ca. 3.5 to 4.5 mA ${\mathrm{cm}}_{\mathrm{disk}}^{-2}$. Figure 5c,d present, in the form of Tafel plots, the specific activity and the mass activity of the tested samples versus the ohmic drop-free applied potential, respectively. As shown in Figure 5c, the highest S.A. was obtained for 7.5 Pd-2.5 Ni_300 at all potentials. The other three electrocatalysts exhibited similar S.A. values in the low overpotential (low current) region, whereas for applied potential higher (less negative) than ca. −0.85 V vs. Ag/AgCl (0.11 V vs. RHE) the S.A. of these electrocatalysts followed the descending order: 7.5 Pd-2.5 Ni_600 > 7.5 Pd-2.5 Ni_200 > 7.5 Pd-2.5 Ni_450. As it concerns the M.A. for HOR (Figure 5d), the following descending order was observed: 7.5 Pd-2.5 Ni_300 > 7.5 Pd-2.5 Ni_450 > 7.5 Pd-2.5 Ni_200 > 7.5 Pd-2.5 Ni_600.

**Figure 6:**
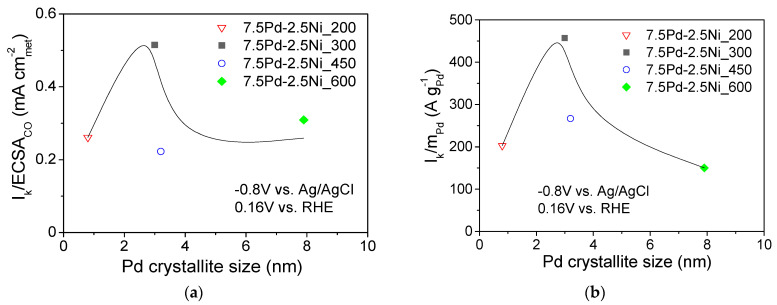
Dependence of the specific activity (**a**) and mass activity (**b**) at −0.8 V vs. Ag/AgCl (0.16 V vs. RHE) on the average Pd crystallite size for the 7.5 Pd-2.5 Ni electrocatalysts that were reduced at 200, 300, 450, and 600 °C.

Figure 6 shows the dependence of the S.A. (Figure 6a) and the M.A. (Figure 6b) at −0.8 V vs. Ag/AgCl (0.16 V vs. RHE) on the Pd average crystallite size for the tested 7.5 wt.% Pd-2.5 wt.% Ni/C electrocatalysts. For Pd crystallite size equal to 2.9 nm, the S.A. (Figure 6a) and the M.A. (Figure 6b) exhibited a maximum. This behavior shows a correlation between the average Pd crystallite size and the HOR electrocatalytic activity, indicating that HOR is a structure-sensitive reaction, in agreement with the conclusions of previous studies of HOR in alkaline medium [29] on Pd/C [1], Rh/C [77], Ru/C [38,41] and Ir/C [40].

The volcano-type dependence of the HOR activity on Pd crystallite size of the tested 7.5 Pd-2.5 Ni electrocatalysts observed in the present study, with the maximum corresponding to ca. 3 nm (for 7.5 Pd-2.5 Ni_300), is similar to that reported by Ohyama et al. [38] for Ru/C and can be partly explained by a surface structure that favors the synergistic effect of HBE decrease on the Pd sites (electronic effect) and OHBE increase on the Ni sites (oxophilic effect). However, the change in reduction temperature may also affect the near-surface atomic composition of the Pt-Ni electrocatalyst for the same bulk composition, similar to the case of Pt-Ru nanoparticles supported on porous carbon [61]. In view of this, the superior activity of 7.5 Pd-2.5 Ni_300 among the tested 7.5 Pd-2.5 Ni electrocatalysts may also be partly explained by an optimum near-surface atomic composition, which results in a balanced adsorption of hydrogen on Pd and of hydroxyl species on Ni that facilitating their reaction in the RDS and, thus, increasing the HOR rate.

### 3.3. Effect of the Pd:Ni Ratio on the HOR Activity of 10 wt.% Pd-Ni/C Electrocatalysts

To investigate the effect of the Pd:Ni ratio on the HOR activity in 0.1 M KOH, a series of Pd-Ni bimetallic electrocatalysts with a 10 wt.% total metal loading and differing Pd:Ni ratio were synthesized via the w.i. method and reduced at 300 °C, which was found to be the optimum reduction temperature for 7.5 wt.% Pd-2.5 wt.% Ni/C (Section 3.2). A 10 wt.% Ni/C electrocatalyst was also synthesized in the same manner to be used as reference material together with 10 wt.% Pd/C. The physicochemical characterization of the synthesized catalyst powders involved the determination of their SSA via $N_{2}$ physisorption (BET method), as well as structural and phase characterization via XRD (Section 2.2).

Table 3 presents the notation, the reduction temperature, the SSA values, and the average Pd crystallite size, as obtained using XRD data for the x wt.% Pd-y wt.% Ni/C and 10 wt.% Pd/C catalysts, as well as the ECSA_CO_ values, as determined using the CO stripping method [54]. The SSA values ranged from 112 m^2^ g^−1^ (6.4 Pd-3.6 Ni) to 155 m^2^ g^−1^ (3.7 Pd-6.3 Ni), in the following descending order: 3.7 Pd-6.3 Ni > 10 Pd > 8.4 Pd-1.6 Ni > 4.7 Pd-5.3 Ni > 7.5 Pd-2.5 Ni > 6.4 Pd-3.6 Ni.

**Figure 7:**
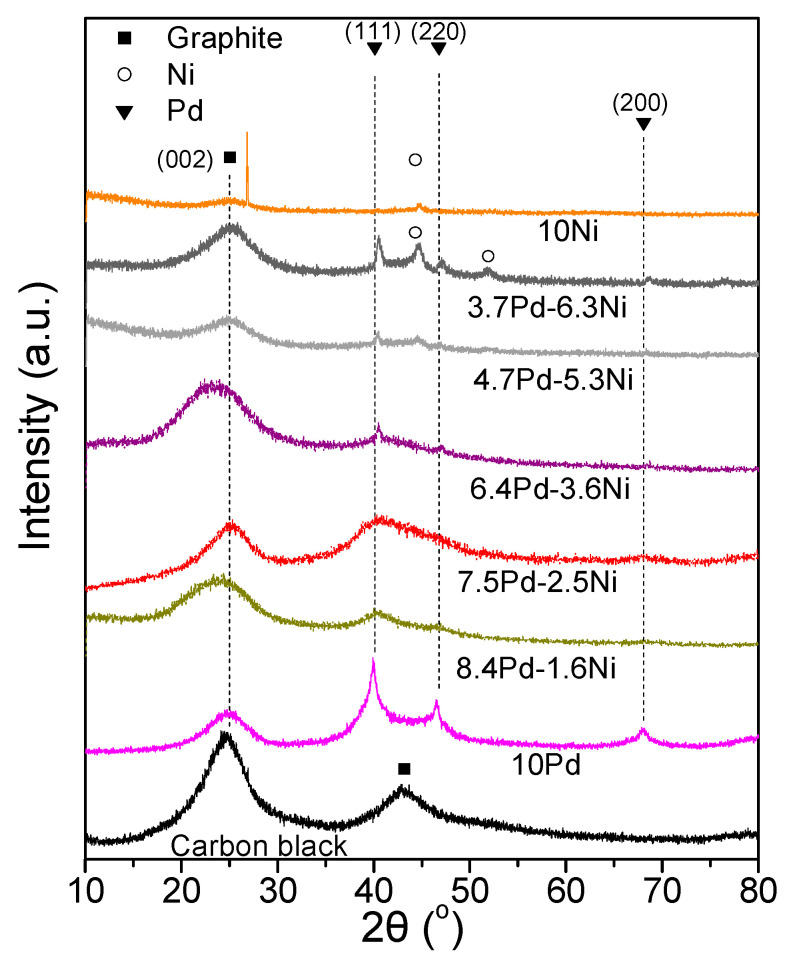
X-ray diffraction patterns of the monometallic 10 Pd and 10 Ni and of the bimetallic x Pd-y Ni catalytic powders with different Pd:Ni atomic ratios (Table 3), reduced at 300 °C, as well as of the carbon black support (Vulcan XC72R).

Figure 7 shows the XRD spectra of the tested samples (Table 3). Alloy formation was not detected in any of the examined catalytic powders. The XRD spectra of the 8.4 Pd-1.6 Ni, 7.5 Pd-2.5 Ni, 6.4 Pd-3.6 Ni, 4.7 Pd-5.3 Ni, and 3.7 Pd-6.3 Ni catalysts were characterized by diffraction peaks located at 2*θ* equal to 40.3°, 46.6°, and 68.0°, attributed to the (111), (200) and (220) facets of the Pd fcc crystalline structure, respectively (JCPDS Card No. 46-1043), for 3.7 Pd-6.3 Ni and 10 wt.% Ni/C (10 Ni) crystallographic peaks attributed to Ni were also detected (JCPDS Card No. 1-1260). Specifically, peaks located at 2*θ* equal to 44.6° and 51.9°, corresponding to the (111) and (200) facets of cubic Ni, were detected in the case of 3.7 Pd-6.3 Ni, whereas only the peak corresponding to the (111) facet was detected in the case of the 10 Ni. The Pd average crystallite size (Table 3) was calculated using the Scherrer equation [53] for the XRD peak located at 2*θ* = 40.3°.

**Figure 8:**
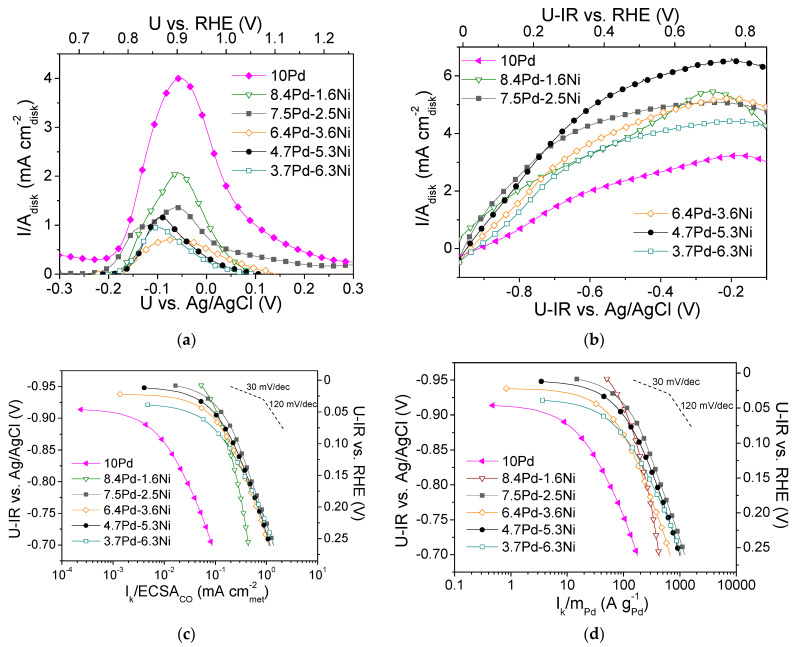
(**a**) CO stripping linear sweep voltammograms obtained in He-purged 0.1 M KOH solution at room temperature with a potential scan rate of 50 mV s^−1^ after previous adsorption of CO at −0.7 V vs. Ag/AgCl for 15 min; (**b**) Polarization curves obtained in 0.1 M KOH solution saturated with $H_{2}$, at room temperature and 3000 rpm, by scanning the potential of the electrocatalyst at a rate of 5 mV s^−1^; Mass-transfer corrected Tafel plots for the HOR in $H_{2}$ saturated 0.1 M KOH solution at room temperature, with the kinetic current *I_k_* normalized with respect to ECSA_CO_ (**c**) and to the Pd mass (**d**) for the monometallic 10 Pd and the bimetallic x Pd-y Ni electrocatalysts with different Pd:Ni atomic ratio, reduced at 300 °C (Table 3). U-IR denotes ohmic drop corrected potential.

The highest ECSA_CO_ value among the Pd-based electrocatalysts (Table 3), as determined from the CO stripping curves shown in Figure 8a, was that for 10 Pd (20.4 m^2^ g^−1^). The addition of nickel in the metal phase resulted in a significant decrease in the ECSA_CO_, which for the Pd-Ni electrocatalysts followed the descending order: 8.4 Pd-1.6 Ni > 7.5 Pd-2.5 Ni > 4.7 Pd-5.3 Ni > 6.4 Pd-3.6 Ni > 3.7 Pd-6.3 Ni, the ECSA_CO_ of 8.4 Pd-1.6 Ni being equal to 20.4 m^2^ g^−1^. With the exception of 3.7 Pd-6.3 Ni, the observed decrease in ECSA_CO_ with a decreasing Pd:Ni ratio was accompanied by an increase of the average Pd crystallite size (Table 3), and could, therefore, be partly attributed to a decrease in the Pd dispersion on the carbon black and a concomitant decrease in the number of available electrocatalytically active sites. However, it should be noted that the determination of ECSA is based on the electrochemical oxidation of CO on both Pd and Ni, so ECSA cannot be correlated exclusively with the Pd crystallite size, especially for the electrocatalysts with high Ni to Pd ratio.

Figure 8b shows the polarization curves for the tested Pd-based electrocatalysts (Table 3), obtained in $H_{2}$-saturated 0.1 M KOH solution at 3000 rpm. Differences in the limiting current density values were observed, with the lowest value being exhibited by 10 Pd (ca. 3 mA ${\mathrm{cm}}_{\mathrm{disk}}^{-2}$) and the highest by 4.7 Pd-6.3 Ni (6.5 mA ${\mathrm{cm}}_{\mathrm{disk}}^{-2}$).

Figure 8c,d shows, in the form of Tafel plots, the specific activity and the mass activity of the Pd-based electrocatalysts, respectively, versus the ohmic drop-free applied potential. As shown in Figure 8c, the highest S.A. was exhibited by the 7.5 Pd-2.5 Ni electrocatalyst. Its superior performance was more pronounced in the low overpotential region. At high overpotentials, its S.A. was similar to that of the 6.4 Pd-3.6 Ni, 4.7 Pd-5.3 Ni, and 3.7 Pd-6.3 Ni electrocatalysts. The 8.4 Pd-1.6 Ni electrocatalyst exhibited lower S.A. compared to the other Pd-Ni bimetallic electrocatalysts in the high overpotentials (high current density) region, while its S.A. appeared to be enhanced at low overpotentials. In terms of M.A. (Figure 8d), practically the same behavior was observed. The 10 Pd electrocatalyst exhibited lower S.A. and M.A. compared to all tested bimetallic x wt.% Pd-y wt.% Ni/C electrocatalysts (Table 3), which highlights the beneficial effect of Ni on HOR activity discussed in the previous sections. It should be noted that the reference monometallic 10 Ni electrocatalyst was practically inactive towards HOR. This can be explained by the fact that hydrogen adsorption on the Ni surface is hindered as it is covered by hydroxyl species due to its oxophilicity [44].

**Figure 9:**
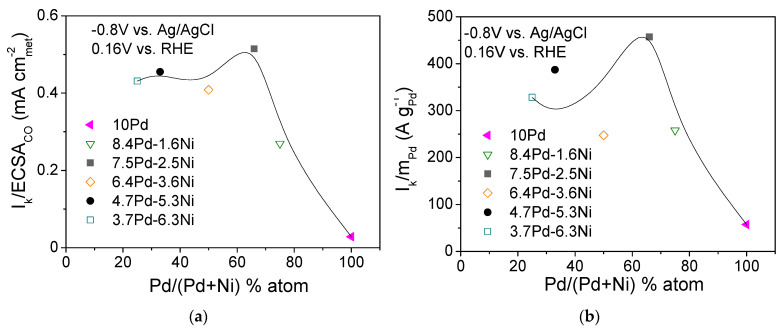
Dependence of the specific activity (**a**) and mass activity (**b**) at −0.8 V vs. Ag/AgCl (0.16 V vs. RHE) on the Pd content for the 10 Pd and the bimetallic x Pd-y Ni electrocatalysts with different Pd:Ni atomic ratio, reduced at 300 °C (Table 3).

Figure 9 shows the dependence of the S.A. (Figure 9a) and M.A. (Figure 9b) at −0.8 V vs. Ag/AgCl (0.16 V vs. RHE) on the Pd content in the metal phase (% atom) for the compared electrocatalysts (Table 3). As shown in the figure, the dependence of the S.A. (Figure 9a) and M.A. (Figure 9b) on the Pd atomic percentage results in a volcano-type plot with a maximum exhibited for the 7.5 Pd-2.5 Ni electrocatalyst. A similar type of behavior was observed by Long et al. [62] in their study of HOR in 0.1 M KOH over Pt-Ru bimetallic nanoparticles of different degrees of alloying. They reported that an increase in the Ru content in the alloy phase from 26 to 35 at.% resulted in a decreasing lattice constant (compared to Pt) and a parallel increase in HOR activity, while further increasing of the Ru content to 38 at.% resulted in a decrease in HOR activity, attributed to phase segregation of Pt and Ru [62].

Overall, on the basis of Figure 9, it could be concluded that the superior performance of 7.5 Pd-2.5 Ni compared to all other tested x wt.% Pd-y wt.% Ni/C electrocatalysts and 10 wt.% Pd/C (Table 3) can be partly associated with a position of the Pd d-band center corresponding to an optimum HBE on the Pd surface, thus accelerating HOR. The presence of a certain Ni content in the metal phase also optimizes the effect of Ni metal oxophilicity, facilitating the adsorption of hydroxyl species on its surface and concomitantly favoring HOR. The Pd-Ni bimetallic system exhibited the highest HOR activity in 0.1 M KOH for a Pd:Ni atomic ratio equal to 2:1 (7.5 wt.% Pd-2.5 wt.% Ni/C) and for reduction temperature equal to 300 °C. For this electrocatalyst (7.5 Pd-2.5 Ni_300), an optimum near-surface atomic composition and structure is presumably obtained, providing a balance between HBE and hydroxyl species adsorption energy and greatly facilitating the reaction between $H_{ad}$ on Pd sites and $OH_{ad}$ on Ni sites in the RDS, which can explain its superior HOR activity among all tested Pd-Ni electrocatalysts.

## 4. Conclusions

In the present work, carbon black-supported 7.5 wt.% Pd-2.5 wt.% M (M: Ag, Ca, Co, Cu, Fe, Ni, Ru, Sn, Zn)/C electrocatalysts were synthesized using the wet impregnation method, physicochemically characterized and compared for their activity for the hydrogen oxidation reaction (HOR) in alkaline medium (0.1 M KOH), using the thin-film rotating-disk electrode technique. 10 wt.% Pt/C, 10 wt.% Pd/C and 10 wt.% Ni/C electrocatalysts synthesized using the same method were employed as reference materials.

The highest HOR activity among the tested samples was exhibited by the monometallic 10 wt.% Pt/C electrocatalyst, while among the Pd-based electrocatalysts it was exhibited by the 7.5 wt.% Pd-2.5 wt.% Ni/C electrocatalyst, followed by 7.5 wt.% Pd-2.5 wt.% Cu/C. The superior HOR performance of 7.5 wt.% Pd-2.5 wt.% Ni/C could be partly related to an optimum Pd lattice strain of ca. 0.65% (strain effect), which corresponds to downshift of the Pd d-band center and concomitant lowering of the HBE on the Pd surface at an optimum value, and the higher oxophilicity of Ni compared to Pd, which favors the adsorption of the hydroxyl species on the Ni surface. The decrease in HBE could also be partly attributed to the electronic interaction between Pd and Ni (ligand effect), which was allowed by some intermixing between Pd and Ni in the bimetallic catalysts, although Pd-Ni alloy formation was not validated. Both the above electronic and oxophilic effects promote the reaction between $OH_{ad}$ on Ni sites and $H_{ad}$ on neighboring Pd sites in the RDS, therefore enhancing HOR activity.

Examination of the effect of the Pd:Ni ratio for 10 wt.% total metal loading and of the reduction temperature on the HOR activity of the Pd-Ni system revealed superior performance for the electrocatalyst with a Pd:Ni atomic ratio equal to 2:1 (7.5 wt.% Pd-2.5 wt.% Ni/C) and reduced at 300 °C.

## Figures and Tables

**Table 1:** Notation, composition, specific surface area (SSA), average metal particle size (TEM), and electrochemically active surface area (ECSA_CO_) of the first series of synthesized catalytic powders, all reduced at 300 °C.

Notation	Composition	SSA (m^2^ g^−1^)	Average Metal Particle Size (nm)	ECSA_CO_ (m^2^ g^−1^)
C	100 wt.% Vulcan XC72R	216 ^1^	-	-
10 Pt	10 wt.% Pt/C	123 ^1^	2.1 ± 0.7 ^1^	15.0 ^2^
10 Pd	10 wt.% Pd/C	143 ^1^	4.0 ± 1.6 ^1^	20.4 ^2^
7.5 Pd-2.5 Ag	7.5 wt.% Pd-2.5 wt.% Ag/C	171 ^1^	5.0 ± 2.0 ^1^	14.0 ^2^
7.5 Pd-2.5 Ca	7.5 wt.% Pd-2.5 wt.% Ca/C	62 ^2^	Not measured	5.0 ^2^
7.5 Pd-2.5 Co	7.5 wt.% Pd-2.5 wt.% Co/C	104 ^1^	Not measured	3.9 ^2^
7.5 Pd-2.5 Cu	7.5 wt.% Pd-2.5 wt.% Cu/C	119 ^1^	5.2 ± 3.4 ^1^	4.4 ^2^
7.5 Pd-2.5 Fe	7.5 wt.% Pd-2.5 wt.% Fe/C	117 ^1^	Not measured	4.7 ^2^
7.5 Pd-2.5 Ni	7.5 wt.% Pd-2.5 wt.% Ni/C	118 ^1^	3.0 ± 0.7 ^1^	6.7 ^2^
7.5 Pd-2.5 Ru	7.5 wt.% Pd-2.5 wt.% Ru/C	125 ^3^	13.0 ± 1.7 ^3^	5.5
7.5 Pd-2.5 Sn	7.5 wt.% Pd-2.5 wt.% Sn/C	141 ^2^	8.5 ± 5.2 ^2^	8.3 ^2^
7.5 Pd-2.5 Zn	7.5 wt.% Pd-2.5 wt.% Zn/C	102 ^1^	5.1 ± 1.9 ^1^	4.9 ^2^

^1^ Ref. [53]. ^2^ Ref. [54]. ^3^ Ref. [52].

**Table 2:** Notation, reduction temperature, SSA, Pd crystallite size (XRD), and ECSA_CO_ of the 7.5 wt.% Pd-2.5 wt.% Ni/C catalyst reduced at four different temperatures.

Notation	Reduction Temperature (°C)	SSA (m^2^ g^−1^)	Pd Crystallite Size (nm)	ECSA_CO_ (m^2^ g^−1^)
7.5 Pd-2.5 Ni_200	200	98	0.8 ± 1.2	5.8
7.5 Pd-2.5 Ni_300	300	118 ^1^	2.9 ± 0.5	6.7 ^2^
7.5 Pd-2.5 Ni_450	450	120	3.2 ± 0.1	9.0
7.5 Pd-2.5 Ni_600	600	134	7.9 ± 0.2	3.6

^1^ Ref. [53]. ^2^ Ref. [54].

**Table 3:** Notation, composition, SSA, Pd crystallite size (XRD), and ECSA_CO_ of the tested 10 wt.% (Pd-Ni)/C and 10 wt.% Pd/C catalysts reduced at 300 °C.

Notation	Composition	Pd:Ni Atomic Ratio	SSA (m^2^ g^−1^)	Pd Crystallite Size (nm)	ECSA_CO_ (m^2^ g^−1^)
10 Pd	10 wt.% Pd/C	-	143 ^1^	3.9 ± 0.9	20.4 ^2^
8.4 Pd-1.6 Ni	8.4 wt.% Pd-1.6 wt.% Ni/C	3:1	141	2.5 ± 0.1	12.3
7.5 Pd-2.5 Ni	7.5 wt.% Pd-2.5 wt.% Ni/C	2:1	118 ^1^	2.9 ± 0.5	6.7 ^2^
6.4 Pd-3.6 Ni	6.4 wt.% Pd-3.6 wt.% Ni/C	1:1	112	4.9 ± 0.3	3.8
4.7 Pd-5.3 Ni	4.7 wt.% Pd-5.3 wt.% Ni/C	1:2	123	22.8 ± 2.1	4.0
3.7 Pd-6.3 Ni	3.7 wt.% Pd-6.3 wt.% Ni/C	1:3	155	16.7 ± 0.7	1.7

^1^ Ref. [53]. ^2^ Ref. [54].

## Data Availability

Data are contained within the article.
